# A novel function of *twins*, B subunit of protein phosphatase 2A, in regulating actin polymerization

**DOI:** 10.1371/journal.pone.0186037

**Published:** 2017-10-04

**Authors:** Po-An Yeh, Ching-Jin Chang

**Affiliations:** 1 Institute of Molecular Biology, Academia Sinica, Nankang, Taipei, Taiwan; 2 Department of Bioscience Technology, Chung Yuan Christian University, Chung Li, Taiwan; 3 Graduate Institute of Biochemical Sciences, College of Life Science, National Taiwan University, Taipei, Taiwan; 4 Institute of Biological Chemistry, Academia Sinica, Nankang, Taipei, Taiwan; University of Dayton, UNITED STATES

## Abstract

Actin is an important component of the cytoskeleton and its polymerization is delicately regulated by several kinases and phosphatases. Heterotrimeric protein phosphatase 2A (PP2A) is a potent phosphatase that is crucial for cell proliferation, apoptosis, tumorigenesis, signal transduction, cytoskeleton arrangement, and neurodegeneration. To facilitate these varied functions, different regulators determine the different targets of PP2A. Among these regulators of PP2A, the B subunits in particular may be involved in cytoskeleton arrangement. However, little is known about the role of PP2A in actin polymerization *in vivo*. Using sophisticated fly genetics, we demonstrated a novel function for the fly B subunit, *twins*, to promote actin polymerization in varied tissue types, suggesting a broad and conserved effect. Furthermore, our genetic data suggest that *twins* may act upstream of the actin-polymerized-proteins, Moesin and Myosin-light-chain, and downstream of Rho to promote actin polymerization. This work opens a new avenue for exploring the biological functions of a PP2A regulator, *twins*, in cytoskeleton regulation.

## Introduction

Protein phosphatase 2A (PP2A) is one of the most prominent serine/threonine phosphatases and reverses the action of kinases in several major cell-signaling cascades [1, 2]. Heterotrimeric PP2A comprises a 36 kDa catalytic subunit (PP2A_C_), a 65 kDa associated subunit (PR65 or A subunit), and a third regulatory subunit, in one of three known classes: PR55/B, PR56/PR61/B', PR72/B'' and PR93/PR110/ B‴ [3–5]. Because there are several isoforms of each component in the mammalian system, it is difficult to analyze the function of each specific subunit. Although many studies utilized selective (but nonspecific) inhibitors in investigating the loss of PP2A function [6], it is more informative to complement the pharmacological results with genetic studies. In *Drosophila*, *twins* is the only B regulatory subunit of PP2A [7, 8], compared to at least four identified isoforms (α, β, γ and δ) in mammals [5]. This simplification makes *Drosophila* an excellent model for the studying of the loss of B subunit function with genetic approaches. In this study, we took advantage of the relatively simplified genetic background of *Drosophila melanogaster* and found a novel role for the B subunit of PP2A in actin polymerization.

B subunits and other PP2A components bind the microtubule cytoskeleton and interact with Tau [9, 10], and furthermore, it has been demonstrated that PP2A is involved in cytoskeleton rearrangement during cell division [11, 12]. Along these lines, the *twins* mutant exhibits abnormal anaphase resolution [7], implying that B subunits might be important in cytoskeleton organization. However, there is no previous experimental evidence *in vivo* that shows the B subunit of PP2A can regulate the cytoskeleton. In *Drosophila*, migration of nuclei is easy to observe in the developing retina, and cell migration is well studied in the border cells of the ovary. Taking advantage of these patterns, we could observe different tissues and found that *twins* was affected the functional readout of the actin polymerization.

Actin polymerization is elegantly regulated by phosphorylation and dephosphorylation events [13], and several kinases and phosphatases are vital in actin polymerization. With regard to PP2A, a limited number of studies have shown that PP2A can bind to and dephosphorylate Cofilin in non-neural cell types, leading to its activation and the severing of F-actin [14, 15]. In this study, we pinpoint the specific PP2A regulation that is involved in the actin polymerization.

The actin cytoskeleton has long been studied in cell culture systems. Here, we took advantage of fly genetics to study physiologically relevant actin dynamics *in vivo*. This approach allowed us to more closely examine actin regulation in a complex living organism. Compared to the cell culture system, it is much more challenging to observe the dynamics of actin cytoskeleton regulation in the living organisms because actin regulation occurs both spatially and temporally. Our model organism was ideal for studying this complex system. *Drosophila* salivary glands are composed of a group of giant cells, in which we can easily detail organelle dynamics. Also, the migration of border cells is a well-studied model system for decoding the molecules involved in actin polymerization [16]. In combination with sophisticated genetic approaches, we were able to analyze selected molecules that interact with or regulate the actin cytoskeleton and compared mutant cells with neighboring control cells *in vivo*. Using these ideas and techniques, we show a novel function of the PP2A regulator, *twins*, involved in actin polymerization.

## Materials and methods

### Fly strains and stocks

*twins*^*60*^ and *twins*^*P*^ were generous gifts from Tadashi Uemura (Graduate School of Biostudies, Kyto University). *capt/acu*^*E593*^ and *UAS-CAPT/ACU* were kindly provided by Jessica Treisman [17], and *en-Ga4*, *UAS-nls-GFP* were obtained from Cheng-Ting Chien (Institute of Molecular Biology, Academia Sinica, Taiwan). *yw*, *hs-FLP; Act5C>y*^*+*^*>Gal4*, *UAS-nls-GFP* [18], *UAS-Dicer2* were gifts from Henry Sun (Institute of Molecular Biology, Academia Sinica, Taiwan). *yw*, *hs-FLP; slbo-GAL4*, *UAS-GFP/CyO* [19] was obtained from Chuen-Chuen Jang (Institute of Biotechnology and Bioindustry Science, National Cheng Kung University). Transgenic flies of *UAS-TWS/twins*, *UAS-twins*^*RNAi*^ and *UAS-hPR55Bβ2* used in this study were generated in previous studies [20, 21].

### Immunoblotting

Adult flies with GMR-Gal4-driven expression of *twins* were collected after two-day eclosion. About 20 adult fly heads were extracted with 100 μL P-TER tissue extraction buffer (Pierce) containing protease inhibitor cocktail (Roche). Protein concentrations from the fly head extractions were measured using the Lowry method (BioRad) before being boiled with 2X sample buffer (2% SDS, 10% glycerol, 0.25 M Tris, 0.01% bromophenol, 5 mM EGTA, 5 mM EDTA, 25 mM DTT, pH 6.8). Each lane was loaded with about one fly head. Immunoblots were stained with monoclonal mouse anti-FLAG and α-tubulin (Sigma; 1:1,000 and 1: 100,000 respectively) for overnight at 4°C. After washing, blots were incubated with peroxidase-conjugated goat anti-mouse (Jackson ImmunoResearch; 1:100,000) diluted in 5% milk powder in TBS with 0.1% Tween 20 for 1 hour at room temperature, and visualized using ECL kits (Millipore). Images were captured by Fujifilm LAS 3000.

### Tissue staining

Virgin female flies with *yw*, *hs-FLP; Act5C>y*^*+*^*>Gal4*, *UAS-nls-GFP* were crossed to male flies with *UAS-TWS*. The progeny were incubated at 37°C for 15 min at the second instar larva stage. Third instar larvae with mosaic green fluorescence were dissected in PBS and tissues were fixed in 3.7% formaldehyde for immunofluorescence staining. Rabbit anti-phospho-Moesin (#3726, 1:200), anti-phospho-MLC (#3671, 1:200) and rabbit anti-phospho-Cofilin (#3313, 1:50) were purchased from Cell Signaling Technology (Beverly, MA, USA). Rabbit anti-MYC (A-14) was purchased from Santa Cruz. Biotechnology (Santa Cruz, CA, USA). Anti-rabbit conjugated with Cy5, used as a secondary antibody, was purchased from Jackson ImmunoResearch. Rhodamine-phalloidin (Invitrogen, Carlsbad, CA, USA) was used to reveal F-actin formation.

### RT-PCR

About 10 fly heads with GMR-Gal4-driven expression of GFP or TWS-Ri were dissected in PBS and extracted with TRIzol (Invitrogen). Total RNA eas precipitated with isopropanol and reverse transcribed with poly-T primer and reverse transcriptase (Invitrogen). Primers used in PCR were 5'-GACCATCCGCCCAGCATACAG-3' and 5'-ATCTCCTTGCGCTTCTTGGAGGAG-3' for the house keeping ribosomal protein RP49, and the TWS primer sequences were 5'-GAGCCCGAATTCGACTACCT-3' and 5'-GCCCTTAGAGCTCGAGTAGA-3'. The PCR conditions were as follows: First denatureation step at 92°C for 2min, followed by 35 cycles of denaturation at 92°C for 30s, annealing at 50°C for 30s, and extension at 72°C for 30s; final extension was performed at 72°C for 7min.

### Scanning electron microscopy

Adult fly heads were dissected in PBS and collected, then dehydrated through an ethanol series and finally transferred to 100% acetone at 4°C overnight before the critical point drying with liquid CO_2_, and sputter-coating with gold. Samples of fly heads were observed and images captured using a JEOL JSM-5600 electron microscope.

## Results

### *twins*, B subunit of PP2A, promotes actin polymerization

**Fig 1:**
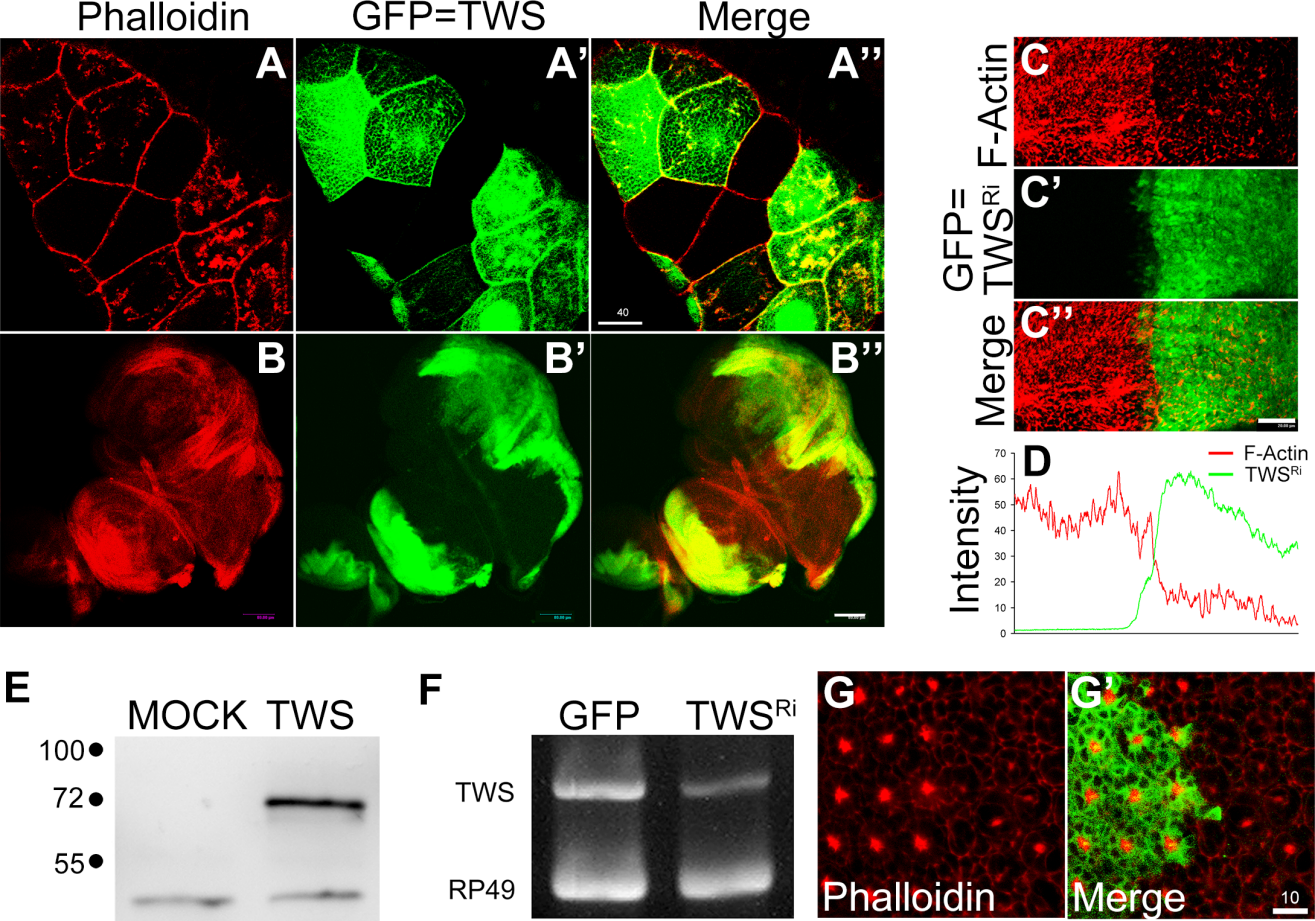
*twins*, B regulator of protein phosphatase 2A promotes actin polymerization. (A-B) Mosaic or partial overexpression of *twins* sustained actin polymerization in both the salivary gland (A) and imaginal discs (B). GFP signals showed where *twins* was overexpressed. (G-D) Double stranded RNA interference (TWS^Ri^) to knockdown *twins* expression diminished actin polymerization. TWS^Ri^ was co-ex-pressed with GFP in the posterior wing pouch. Further densitometric analysis demonstrated that as *twins* was depleted, actin polymerization declined. (E) Western blotting analysis using FLAG antibody revealed the GMR-Gal4 expression of FLAG-tagged TWS. (F) RT-PCR showed the *twins* knockdown effect, where RP49 was the control. (G) In the absence of GFP, which represented the *twins* mutant clones, ommatidia showed loss of rhabdomere structures. Genetic background: (A) *yw hsFLP*; *act>y+>GAL4*, *UAS-GFP/UAS-TWS*. (B) *en-GAL4/+*; *UAS-GFP/UAS-TWS*. (C, D) *en-GAL4/+*; *UAS-GFP /UAS-TWS*^*Ri*^. (E) *GMR-GAL4>FLAG-TWS*. (F) *GMR>TWS-Ri*. (G) *yw hsFLP*; *FRT82B twins*^*60*^*/FRT82B ubi-GFP*. Scale bars (μm), 40(A”), 80(B”), 20(C”), 10(G’).

Although previous studies suggested that PP2A plays a role in cytoskeleton regulation [22–25], they did not provide a detailed picture of the underlying mechanism. Here, by using *Drosophila* as a model organism, we investigated the biological function of a specific regulatory subunit in actin polymerization. Among several PP2A regulatory subunits, we found that *twins*, the B regulatory subunit of PP2A, remarkably elevated actin polymerization in the salivary gland as revealed by phalloidin staining (Fig 1A-1A”), where *twins* was clonally over-expressed by ‘flip-out’ genetic manipulation [18]. The occurrence was 100% penetrance (72 *twins* positive clonal cells). To confirm that this phenomenon was brought about by *twins* overexpression, wing imaginal discs of wandering larvae were used to conduct the same experiment. Consistent with salivary gland results, using *en-GAL4* to over-express *twins* in the posterior half portion of the wing and leg discs robustly increased actin-polymerization compared the non-expressed anterior portion (Fig 1B). The occurrence was also full penetrance in 31 examined wing discs. Furthermore, a double-strained RNA interference knockdown of *twins* (achieved by making transgenic flies carrying UAS vector [26], resulting in substantially decreased actin polymerization (Fig 1C and 1D). The occurrence was 64% (in 29/45 examined wing discs). The overexpression or knockdown effects of *twins* were further examined and confirmed using western blot and RT-PCR, respectively (Fig 1E and 1F).

All these data indicate that *twins* plays a role in actin polymerization. To further investigate the function of endogenous *twins* in actin polymerization, we investigated whether there is any morphological change in the rhabdomere, a *Drosophila* compound eye structure very rich in actin-polymerization structures. Performing a mosaic mutant clone analysis using loss-of-function allele [27], we observed that the rhabdomere, a structure rich in F-Actin [28], was severely diminished in the each ommatidia when the function of *twins* was completely depleted (Fig 1G). The occurrence was 100% penetrance in larger *twins* mutant clones. However, in the small *twins* mutant clones, the rhabdomere still remained intact, suggesting the cytosolic contribution of *twins* can sustain function during the cell divisions. We observed these phenomena in a range of tissue types, suggesting that *twins* regulation of actin polymerization occurs widely throughout the organism.

### *twins* promotes the phosphorylation of Moesin, MLC and Cofilin

**Fig 2:**
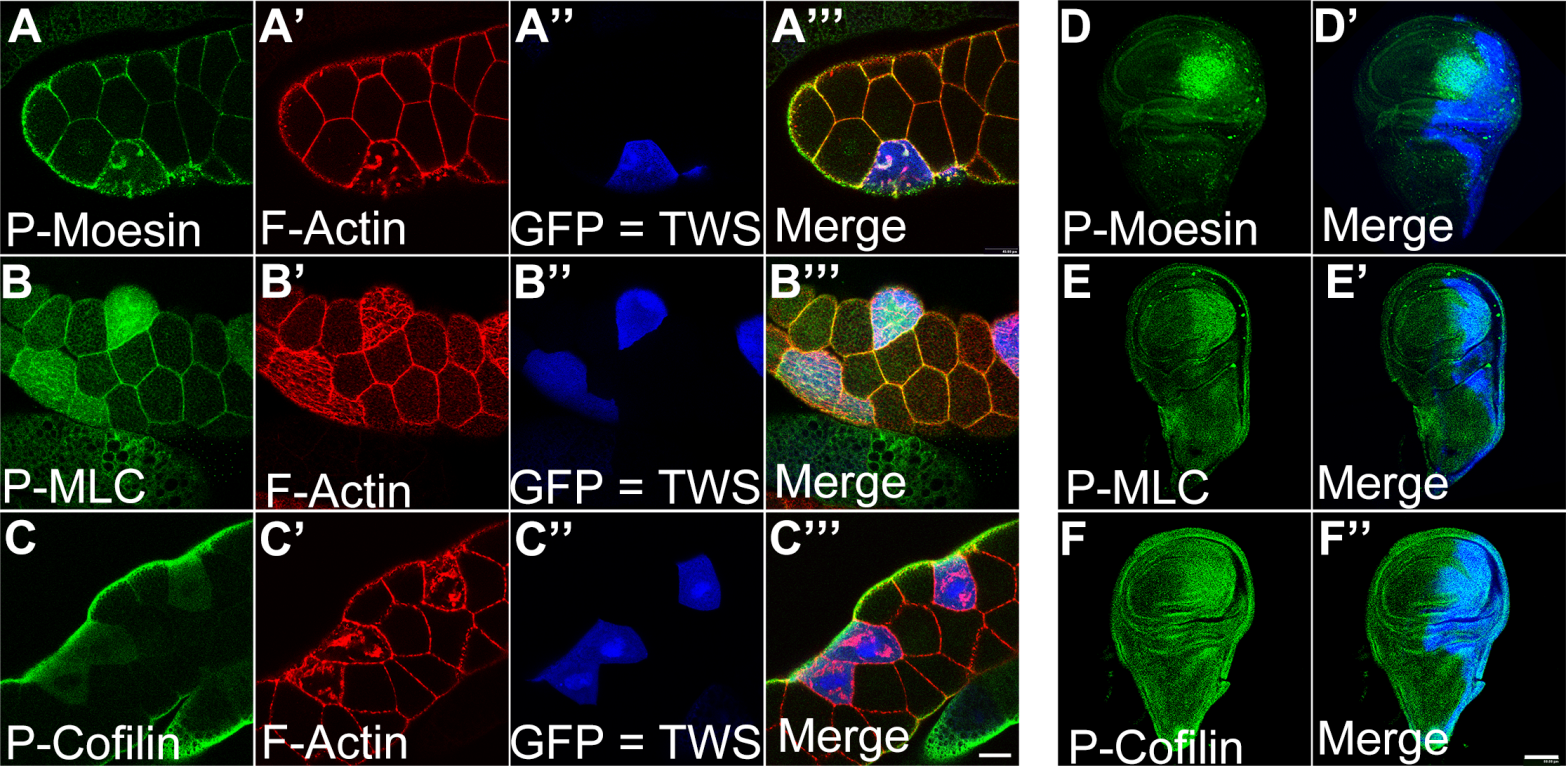
Overexpressing *twins* elevates phosphorylated Moesin, MLC, and Cofilin. (A-C) Salivary glands were dissected from third instar larvae and stained with various antibodies (green in A, B, C) recognizing phospho-proteins that promote actin polymerization, as indicated; phalloidin (red in A’, B’, C’) is a marker for filamentous actin; GFP (blue pseudocolor in A”, B”, C”) shows the cells overexpressing *twins*. (D-F) Wing discs of third instar larvae were stained with phospho-antibodies, as indicated (green in D, E, F); GFP (blue pseudocolor in D’, E’, F’) showed the posterior part of the wing overexpressing *twins*. Genetic background: (A-C) *yw hsFLP*; *Act>y+>GAL4*, *UAS-GFP/UAS-TWS*. (D-F) *en-GAL4*, *UAS-GFP/UAS-TWS*. Scale bars, 40 μm (C”‘), 80μm (F’).

Because actin polymerization increased as *twins* was overexpressed and decreased as *twins* was withdrawn, we further examined Cofilin activity of the downstream protein regulating F-actin formation and the F-actin relevant structure proteins, including Moesin and Myosin light chain (MLC). Phosphorylated Moesin and MLC are the active forms sustaining actin cytoskeleton organization. In contrast, phosphorylated Cofilin, an actin depolymerization protein severing F-actin, is an inactive form allowing unrestricted actin polymerization [29]. In this study, with random or partial overexpression of *twins*, we found that both Moesin and MLC were more phosphorylated compared to the normal cells in salivary glands (Fig 2A and 2B). Furthermore, the same approach revealed that the actin depolymerization factor, Cofilin, was also phosphorylated and inactivated in the cells overexpressing *twins* (Fig 2C). The wing discs had similar results with partial overexpression of *twins* using *en-GAL4* (Fig 2D–2F). However, we noted that the increase in phalloidin staining did not completely match the boundary of induction clones, suggesting that *twins* might have a non-autonomous effect on actin polymerization. These findings suggest that *twins* is involved in promoting the phosphorylation of actin-regulated proteins, Moesin, MLC, and Cofilin. In the 30 samples for each experiment, the occurrences were 100% penetrance.

### Knockout of *twins* impairs neuronal nuclear migration

**Fig 3:**
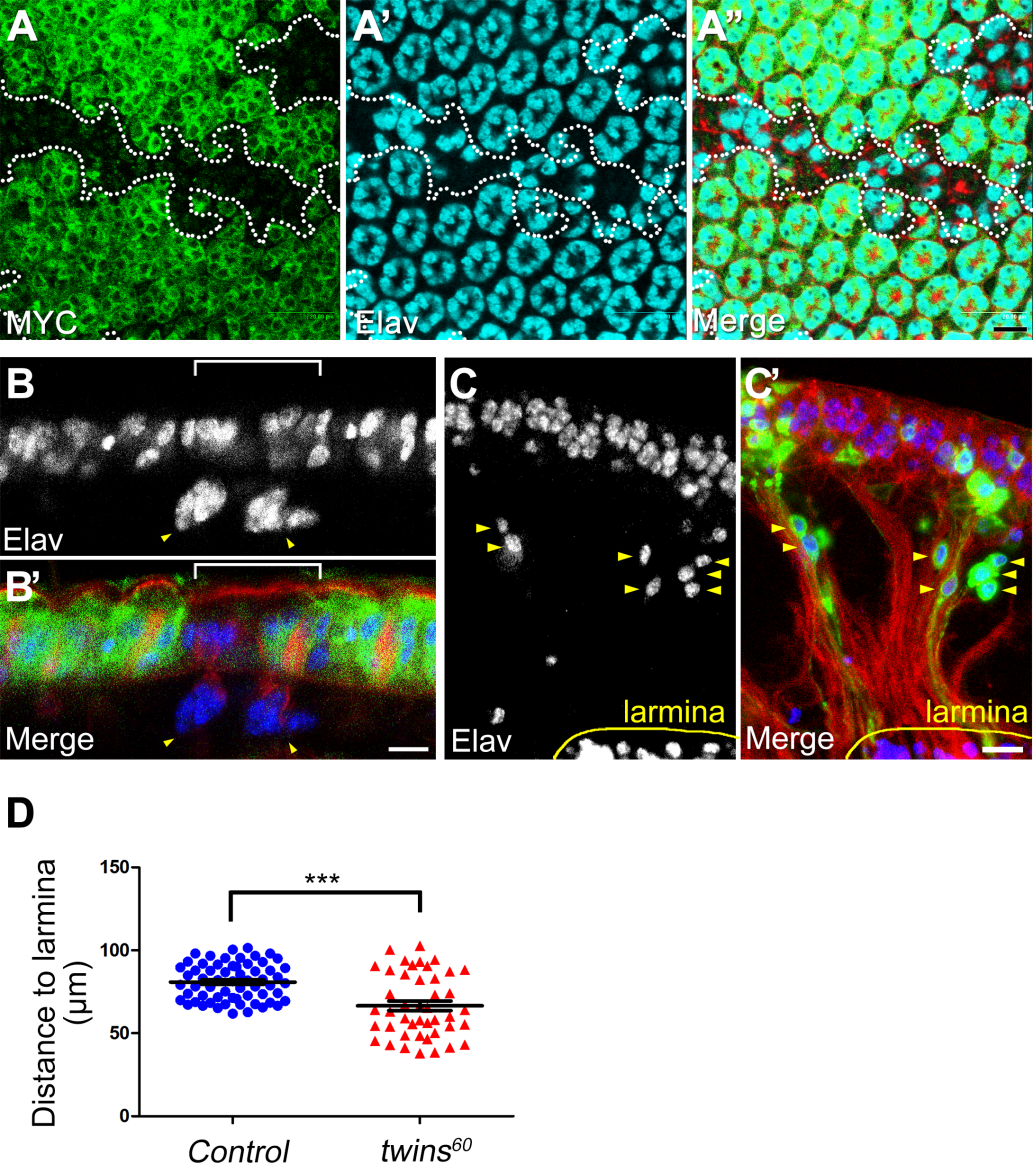
Depletion of *twins* impairs neuronal nucleus migration. (A-B’) Crosscutting (A, A’) and longitudinal view (B, B’) of the pupal retina shows that cells without *twins* function cannot completely reach the normal position. Staining cells with loss of MYC staining (green), which represent *twins* mutant clones (dash line marked or basket), shows impaired neuronal nuclear (blue or cyan: Elav) migration (arrowhead). (C, C’) *twins* MARCM mutant clones with GFP marker show impaired nuclear migration (arrowhead). Red: phalloidin. Scale bars, 10 μm. (D) Statistic analysis of (C) shows the distances between retinal nuclei between lamina. (Mean ± SEM; ***P<0.0001 by Student’s *t*-test) Genotype: (A-B) *yw hsFLP*; *FRT82B twins*^*60*^*/FRT82B N-MYC*. (C) *Elav-GAL4*, *UAS-mCD8GFP*, *hsFLP*; *FRT82B twins*^*60*^*/ FRT82B tub-GAL80*.

**Fig 4:**
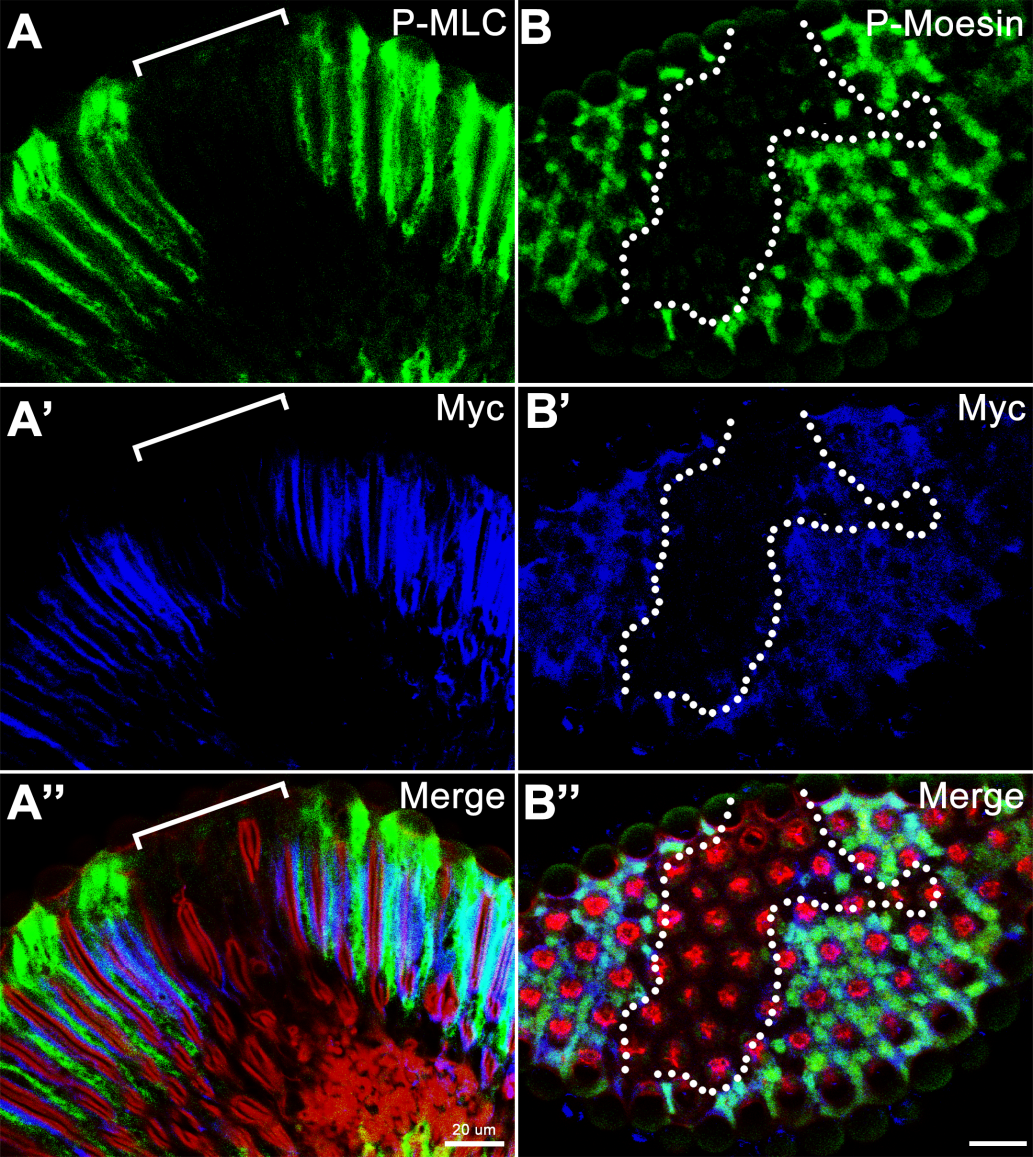
Depletion of *twins* decreases phosphorylated Moesin and MLC in retina. (A-A”) Longitudinal view of the pupa eye shows the staining with P-MLC in green, MYC in blue (*twins* positive cells) and phalloidin in red. (B-B”) Cross section view of the pupa eye shows the staining with P-Moesin in green, MYC in blue (*twins* positive cells) and phalloidin in red. Scare bars, 20 μm. Genotype: *yw hsFLP; FRT82B twins*^*60*^*/FRT82B N-MYC*.

Dynamic actin polymerization is one of the seminal events for cell migration and motility. Arising from our prior observations indicating that loss of *twins* function was accompanied by impairment of actin polymerization, we investigated the biological function of *twins* in cell migration. Given that the neuronal nuclei of *Drosophila* photoreceptor neurons are an ideal system to observe nucleus migration [17], we used somatic recombination to completely deplete the function of *twins* and found that the neuronal nuclear migration was remarkably decelerated (Fig 3A–3B’). To further confirm *twins* function in the nuclear migration of photoreceptor neurons, we utilized an alternative approach, the MARCM genetic technique [30], to see whether nuclear migration was still impaired. Consistent with somatic recombination, the nuclei of the photoreceptor neurons marked with GFP (representing *twins* mutant cells) showed significantly slower migration below the regular photoreceptor layer (Fig 3C and 3D). Further examination of actin regulators by mosaic clonal analysis demonstrated that phosphorylated MLC and Moesin were both decreased in the *twins* mutant background (Fig 4A–4B”) (100% penetrance in 32 clones), supporting our hypothesis that the B subunit of PP2A, *twins*, is involved in actin polymerization and affects nuclear migration.

### Loss of *twins* function impairs border cell migration and nurse cell cytoplasmic dumping

**Fig 5:**
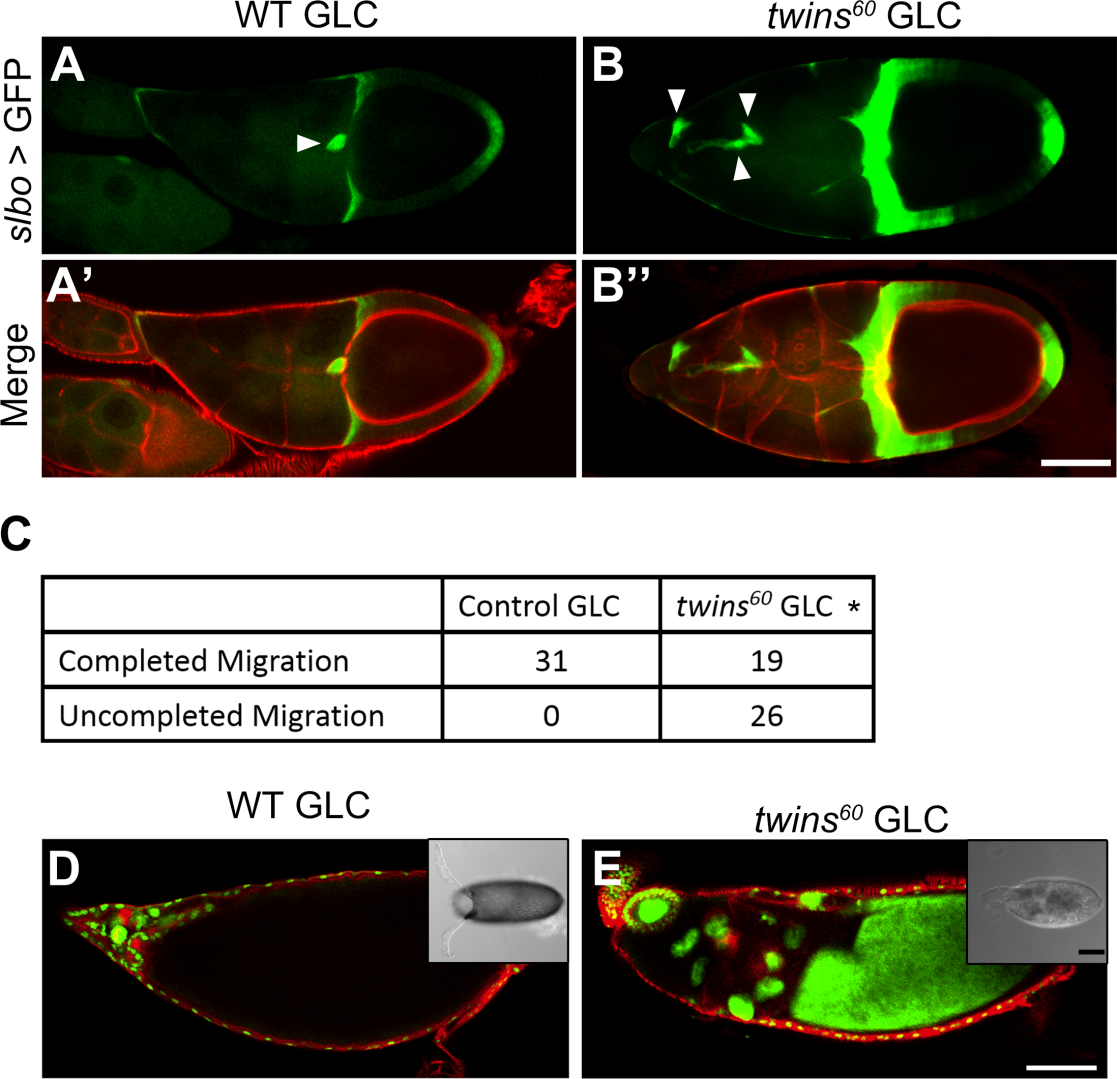
Depletion of *twins* function impairs ovarian border cell migration and cytoplasm dumping. (A, A’) Stage 10b of wild type GLC ovary showed that border cells (white arrowhead) migrated to the edge of the oocyte. (B, B’) *twins* mutant GLC ovary appeared to slow border cell migration. Green represents border cells; red shows phalloidin staining. (C) Table shows statistical analysis. *p*<0.05 by Fisher’s exact test analysis. (D, E) *twins* mutant nurse cells were incapable of dumping the cytosol into the oocyte and still occupied the anterior space of the egg. Green shows nuclear staining by sytox; red for phalloidin. Insert B&W images show abnormal egg morphology of *twins* GLC with shorter egg length and malformed appendages compared to the wild type GLC (E). Genotype: (A, D) *yw*, *hsFLP*; *slbo-GAL4*, *UAS-GFP; FRT82B tub-β-gal/FRT82B OVO*^*D1*^. (B, E) *yw*, *hsFLP*; *slbo-GAL4*, *UAS-GFP; FRT82B twins*^*60*^*/FRT82B OVO*^*D1*^. Scale bar, 80 μm (B”, E), 100 μm (E insert).

To further investigate the role of *twins* in the cell migration of other non-neuronal tissues, we measured the migration of border cells, which normally migrate from the anterior to the middle of the ovary [31]. Several studies have demonstrated that once actin organization or polymerization is impaired, the border cell migration will be affected [32–34]. Along these lines, border cell migration under the *twins* null background (achieved by germ-line-clone (GLC) genetic manipulation [35]), appeared to be markedly impaired (Fig 5A–5C). Interestingly, in the *twins*-null GLC, we also observed that the cytoplasm dumping of nurse cells to the oocyte was severely blocked, resulting in small egg size (Fig 5D and 5E). These observations point to the vital importance of *twins* function for cell migration, which is achieved by regulating actin polymerization in both neuronal and non-neuronal cells.

**Fig 6:**
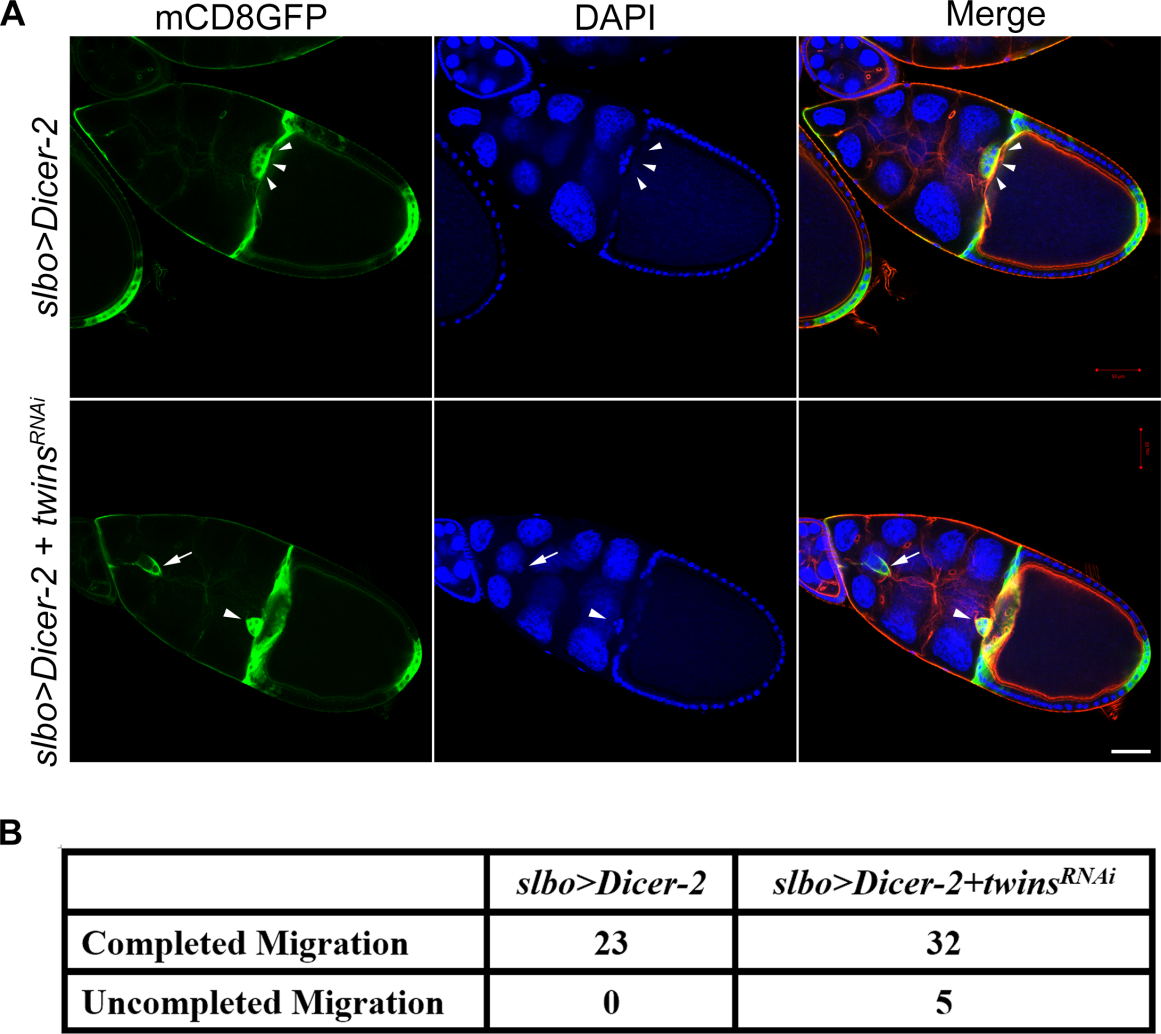
Specific knockdown of *twins* in border cells affects migration. (A) Combination with Dicer-2 to enhance RNA interference of *twins* in border cells resulted in slow cell migration. White arrowheads indicate the correct position of border cells. Arrow indicates the slowed border cell migration as *twins* was depleted. Each fluorescence color indicates: green: mCD8GFP, blue: DAPI, red: phalloidin. Scale bar, 50 μm. (B) Statistic table of the number of border cell completed cell migration. *p* = 0.0798 by Fisher exact test.

To further clarify whether the migration defect of border cells in the *twins* mutant background was cell autonomous or nonautonomous, we performed specific *twins* knockdown experiments in the border cells. By using Dicer-2 [36] to enhance the knockdown effect of *twins*, we observed that border cell migration was affected (Fig 6A). However, the penetrance was not as high as *twins* null GLC (Fig 6B). This discrepancy may be due to uncompelled knockdown of *twins*, or there could be a nonautonomous effect of *twins* exerted from the surrounding nurse cells. Therefore, *twins* depletion in border cells might be not sufficient to generate high penetrance of impaired border cell migration.

**Fig 7:**
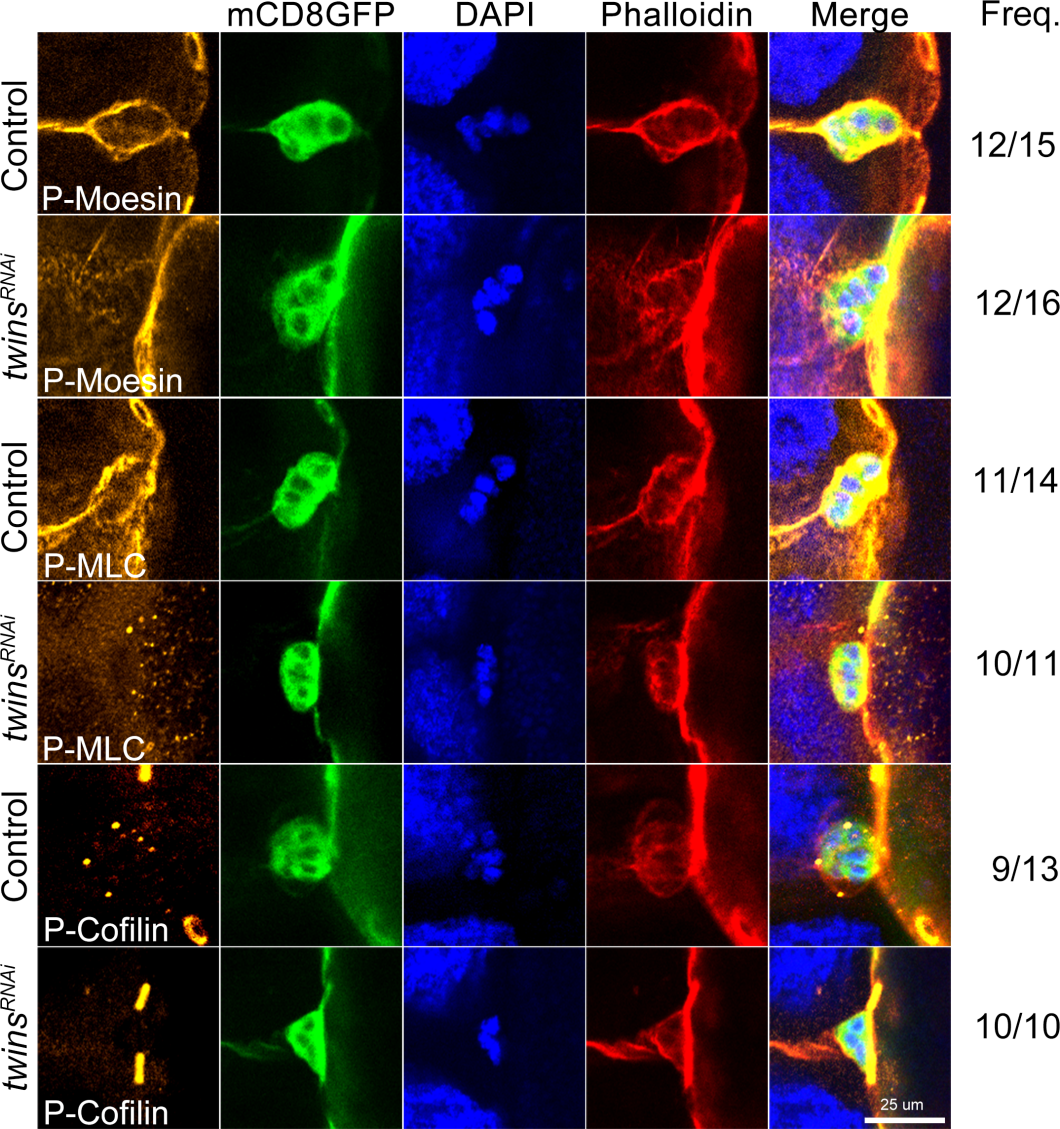
Decreasing phosphorylation of Moesin, MLC, and Cofilin in border cells without *twins* function. Around stage 9–10, ovaries were collected and stained with phosphor-specific antibodies as indicated (orange). Images of green show mCD8GFP, blue for DAPI, red for phalloidin. Each frequency (Freq.) is attached to the right. Scale bar, 25 μm. Genotype: Control, *slbo>mCD8GFP + Dicer-2*; *twins*^*RNAi*^, *slbo>mCD8GFP + Dicer-2 + twins*^*RNAi*^.

We then examined the phosphorylation status of Moesin, MLC and Cofilin in these border cells with loss of *twins* function, conducting the antibody staining with the same knockdown approach. Phospho-specific antibodies revealed that the phosphorylation status of Moesin, MLC, and Cofilin was decreased in the border cells alongside *twins* depletion (Fig 7). These results are consistent with our observation in the salivary glands, retinas, and wing discs.

### *twins* acts downstream of Rho to promote actin polymerization

**Fig 8:**
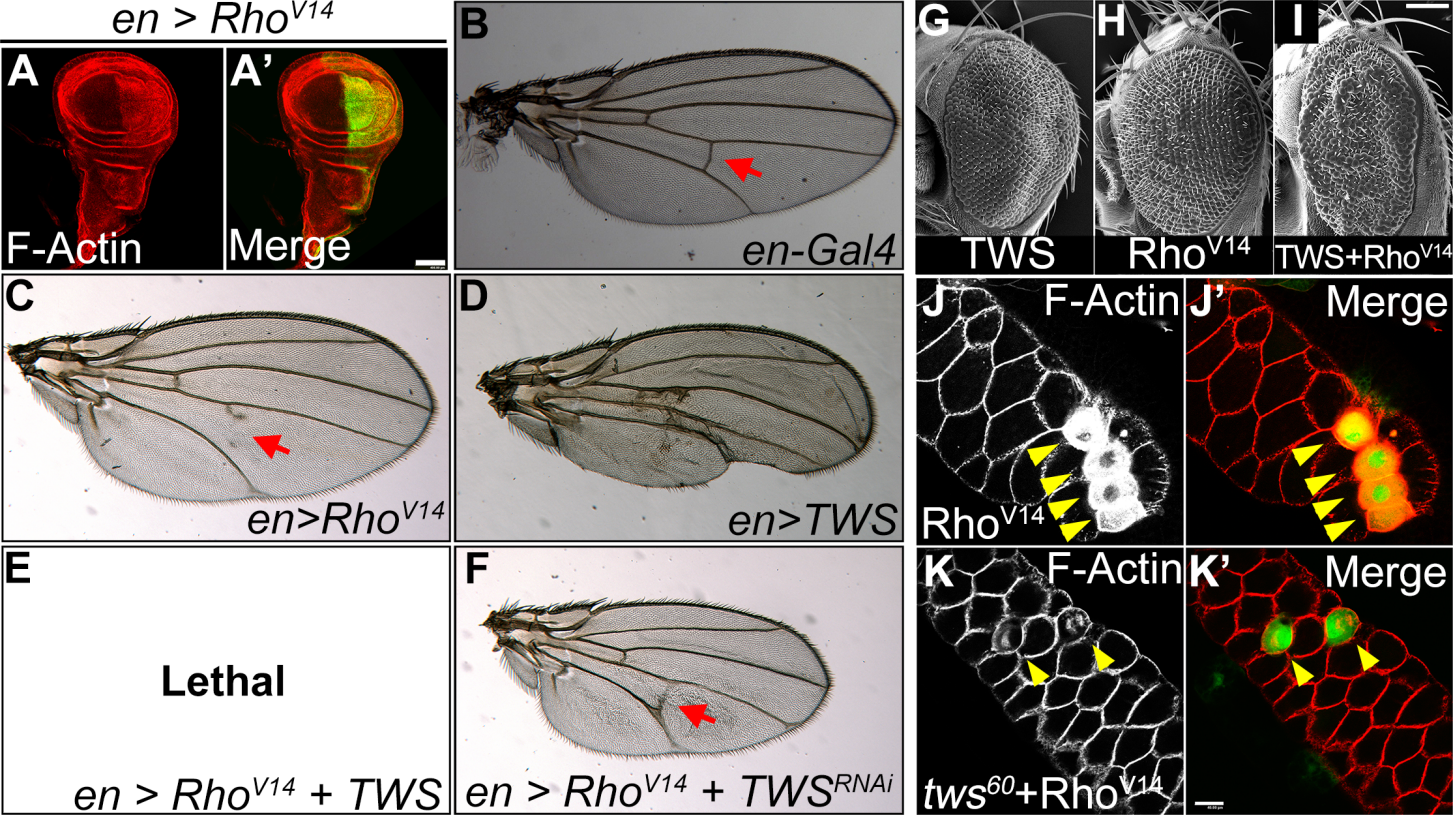
*twins* plays a downstream role of Rho to activate actin polymerization. (A) The expressing dominant active Rho (Rho^V14^) sustained actin polymerization. (B-F) Adult wing phenotypes of genetic interaction between Rho and *twins*. Expressing Rho^V14^ resulted in cross wing vein loss (red arrow in C). (D) Overexpression of *twins* resulted in deformed wing. (E) Co-expression of Rho^v14^ and TWS turned out to be lethal. (F) The phenotype of wing vein loss caused by Rho^V14^ expression could be rescued by the depletion of *twins*. (G-I) Coexpressing TWS with Rho^V14^ in the eye also enhanced the rough eye phenomena. (J) Expression of Rho^V14^ elicited severe actin polymerization (yellow arrowheads). (K) Removal of *twins* function mitigated actin polymerization induced by Rho^V14^ expression. The red color indicates phalloidin staining, and green color shows the cells expressing Rho^V14^. Genotype: (A) *en-GAL4*, *UAS-GFP/UAS-Rho*^*V14*^. (B) *en-GAL4/+* (C) *en-GAL4/UAS-Rho*^*V14*^. (D) *en-GAL4/UAS-TWS*. (E) *en-GAL4*, *UAS-TWS/UAS-Rho*^*V14*^. (F) *en-GAL4*, *UAS-TWS*^*RNAi*^*/UAS-Rho*^*V14*^. (G) *GMR-GAL4/UAS-TWS*. (H) *GMR-GAL4/UAS-Rho*^*V14*^. (I) *GMR-GAL4*, *UAS-TWS/ UAS-Rho*^*V14*^. (J) *yw hsFLP*; *Act>y+>Gal4*, *UAS-GFP/UAS-Rho*^*V14*^. (K) *yw hsFLP*; *Act>y+>GAL4*, *UAS-GFP/UAS-Rho*^*V14*^*; twins*^*60*^*/ twins*^*60*^. Scale bar, 40 μm (A’), 100 μm (K’).

To determine the molecular mechanism of actin polymerization involving *twins*, we examined the genetic interaction of *twins* with several actin regulators. Among these, Rho, a small GTPase, has been shown to play an upstream role in actin polymerization [37]. For this reason, we first investigated the genetic relationship between *twins* and Rho in the actin polymerization pathway. To demonstrate that a dominant active Rho fly strain, UAS-Rho^V14^, functions to promote actin polymerization, we first expressed Rho^V14^ in the posterior wing discs by *en-GAL4*. As expected, this resulted in the increase of polymerized actin (Fig 8A), and it also produced a marked lost-wing-vein phenotype (Fig 8B and 8C). Overexpression of *twins*, which promotes actin polymerization, led to a deformed wing (Fig 8D). When *twins* was co-expressed with Rho^V14^, the combination turned out to be lethal with *en-GAL4* driver expression. It is worth noting that, when we conversely depleted *twins* and expressed Rho^V14^, the wing-vein-lost phenotype was reduced (Fig 8E and 8F). These data suggest that *twins* functions down stream of Rho to promote actin polymerization.

To further verify this mechanism, we performed similar genetic experiments on the eye and salivary gland tissues. Consistent with our previous results, *twins* coexpression with Rho^V14^ in the retina enhanced the rough eye phenotype (Fig 8G–8I). Conversely, loss of *twins* function substantially ameliorated actin polymerization raised by Rho^V14^ expression in the salivary gland (Fig 8J–8K’). These observations confirm that *twins* acted downstream of Rho to promote actin polymerization in a broad range of tissues.

### Malformed rhabdomere structure caused by overexpression of PP2A-B could be restored by *capulet/actin-up*

**Fig 9:**
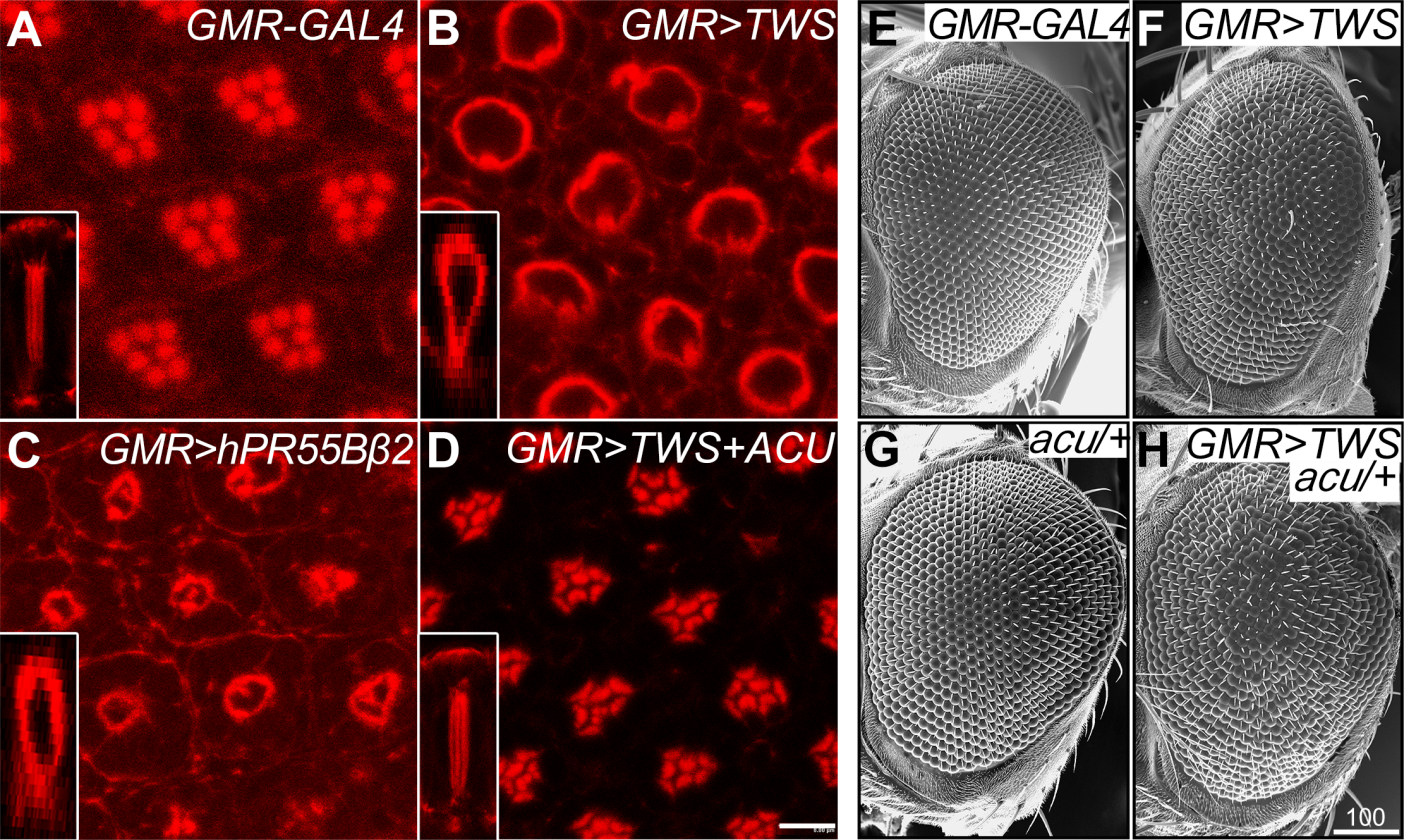
*capt*/*acu* rescues malformed rhabdomere caused by PP2A-B overexpression. (A-D) Eyes dissected from 90% developed pupae were stained with phalloidin. (A) Control pupa eye shows seven compact and distinctly arranged rhabdomere structures. (B) Overexpression of *twins* disrupted rhobdomere structure, causing the circled structures. (C) Expressing a human B subunit of PP2A (*hPR55Bβ2*) leads to the similar disorganized rhabdomere structures. (D) Co-expression with *capt*/*acu* ameliorated deformed rhabdomere structures resulting from PP2A-B overexpression. Each left lower inset shows a lateral view of the rhabdomere structure. (E-H) SEM photos of adult fly eyes. (E) Control eye phenotype. (F) Rough eye phenotype from *twins* overexpression. (G) Removing one copy of *capt*/*acu* alone did not disrupt eye morphology. (H) Overexpression of *twins* in the absence of one copy of *acu* enhanced the rough eye phenotype. Genotype: (A, E) *GMR-GAL4/+* (B, F) *GMR-GAL4/UAS-TWS*. (C) *GMR-GAL4/ UAS-hPR55Bβ2*. (D) *GMR-GAL4*, *UAS-TWS/UAS-ACU*. (G) *GMR-GAL4/+; acu/+*. (H) *GMR-GAL4*, *UAS-TWS/acu*. Scale bar, 8 μm (D), 100 μm (H).

The rhabdomere of photoreceptor neurons is a structure full of F-actin and can be identified by compact phalloidin staining and transmission electron microscopy [38]. Fly rhabdomere structure morphology has long served as a tool to evaluate the neurotoxicity of numerous human pathological genes [39]. Our prior data showing the absent rhabdomere structure in the *twins* mutant clones imply that *twins* might play a potent role in the formation of the rhabdomere. Therefore, we further examined the effect of *twins* gain-of-function in the rhabdomere structure. It is worth noting that *twins* overexpression in the retina severely disrupted the compact rhabdomere structure, and circular rhabdomere shapes formed with the adjacent photoreceptor neurons (Fig 9A and 9B). Furthermore, when we expressed a human B subunit ortholog, *hPR55Bβ2*, we observed similar malformed rhabdomere structures (Fig 9C). These results suggest the functional conservation and similarity between the human *hPR55Bβ2* and fly *twins*. Importantly, coexpression of *twins* with the actin-depolymerization protein, *capulte*/*actin-up* (*capt*/*acu*) [17, 29], rescued the deformed rhabdomere structures (Fig 9D). On the other hand, the rough eye phenotype caused by *twins* overexpression was enhanced under a *capt*/*acu* heterozygous mutant background (Fig 9E–9H). The occurrence was 100% penetrance in 50 examined retinas. Taken altogether, it seems the malformed rhabdomere structure of the photoreceptor neurons arising from *twins* or *hPR55Bβ2* overexpression was associated with the promotion of actin polymerization, and this function of *twins* in the regulation of actin polymerization is upstream of *caput*/*acu*.

## Discussion

By using the genetic technique of mosaic clone analysis, we clearly showed that the specific regulatory subunit *twins* of PP2A could promote actin polymerization, and, conversely, that the loss of this regulator impaired actin polymerization. These findings also demonstrated how suitable the salivary gland and wing disc are for observing actin polymerization *in vivo*. Furthermore, the role of *twins* in actin polymerization was proved to be downstream of Rho and upstream of *capt*/*acu*. Based on our results, we propose two future lines of research to gain further insight. First, the role of several mammalian *twins* orthologs including α, β, γ and δ, has not yet been clearly characterized [5]. In this study, we only tested the expression of the human Bβ2 regulator, whose expression led to an aberrant rhobdomere phenotype similar to *twins* overexpression. However, expression of human Bβ2 regulator in other tissues (e.g., wing and salivary gland) did not generate any visible phenotype of actin polymerization (data not shown), suggesting that *twins* plays more roles than dose Bβ2 dose. On the other hand, the predominant B regulatory pathway in mammals is Bα, which has been shown to be involved in tumor formation [40]. It will be interesting to see the effect of this regulator Bα on actin polymerization. Secondly, although we have shown that overexpression of *twins* elicited actin polymerization, we have not yet identified whether other PP2A catalytic activity is required. Interestingly, a previous study showed that depletion of the PP2A catalytic subunit affected chloroplast migration through the regulation of actin-depolymering-factor (ADF)/cofilin [24]. It will thus be a significant finding to identify the substrate of *twins* in promoting actin polymerization.

In prior research, the activities of kinases and phosphatases, such as LIM-domain kinase, TES kinase, ROH-associated protein kinase, Chronophin and Slinghshot 1, have been largely shown to be involved in actin polymerization [41]. In this study, we found that LIMK [42] did not exert the same rhabdomere phenotype as *twins*, although LIMK was found to promote actin polymerization in the salivary gland and wing discs (S1 Fig). These differences indicate that distinct actin polymerization is regulated in specific tissues. In this light, the current study suggest a new perspective on PP2A regulator, *twins*, regulating actin polymerization.

In the ovary, border cell migration was severely affected under a *twins* mutant background. However, it is unclear whether the surrounding nurse cells also contributed to the effect on border cell migration. An RNA interference (RNAi) approach might help to untangle this puzzle. However, given that *twins* sustains a very strong maternal effect, it could be difficult to apply a knockdown strategy to study the mutant phenotype of *twins*. With regard to PP2A activity, we overexpressed the catalytic subunit of PP2A, MTS [43], but did not see elevation of actin polymerization (data not shown). This suggests that other regulator subunits are involved in the actin polymerization, in opposition to *twins* function. For instance, it has been reported that PP2A binds to Cofilin and severs the actin [44], showing the opposite regulation of actin polymerization to *twins*. However, the specific subunit directing PP2A activity to Cofilin has not yet been reported. Another possibility is that the role of *twins* in regulating actin polymerization is independent of PP2A activity. In light of our data, with or without PP2A activity, *twins* plays vital roles in actin polymerization.

## Supporting information

S1 Fig Dlimk increases actin polymerization, but not leads to rhabdomere disorganized. (A, B) Overexpression of Dlimk markedly raised the level of polymerized actin, revealed by random expression in the salivary gland or *en-GAL4* in the wing disc. (C) Overexpressing Dlimk in eye did not disrupt the regular organization of rhabdomere. Green labeled cells represented GFP expression with Dlimk. Red florescence shows phalloidin staining. Scale bar, 40 μm (A”, B”), 8μm (C). Genetic background: (A) *yw hsFLP*; *act>y+>GFP/ UAS-Dlimk*. (B) *en-GAL4*, *UAS-GFP/ UAS-Dlimk*. (C) *GMR-GAL4/ UAS-Dlimk*.
(TIF)
