# Supplementary figures and images for: A novel function of twins, B subunit of protein phosphatase 2A, in regulating actin polymerization
Source: PLoS One. 2017 Oct 4;12(10):e0186037. doi: 10.1371/journal.pone.0186037 (PMC5627941; doi:10.1371/journal.pone.0186037)

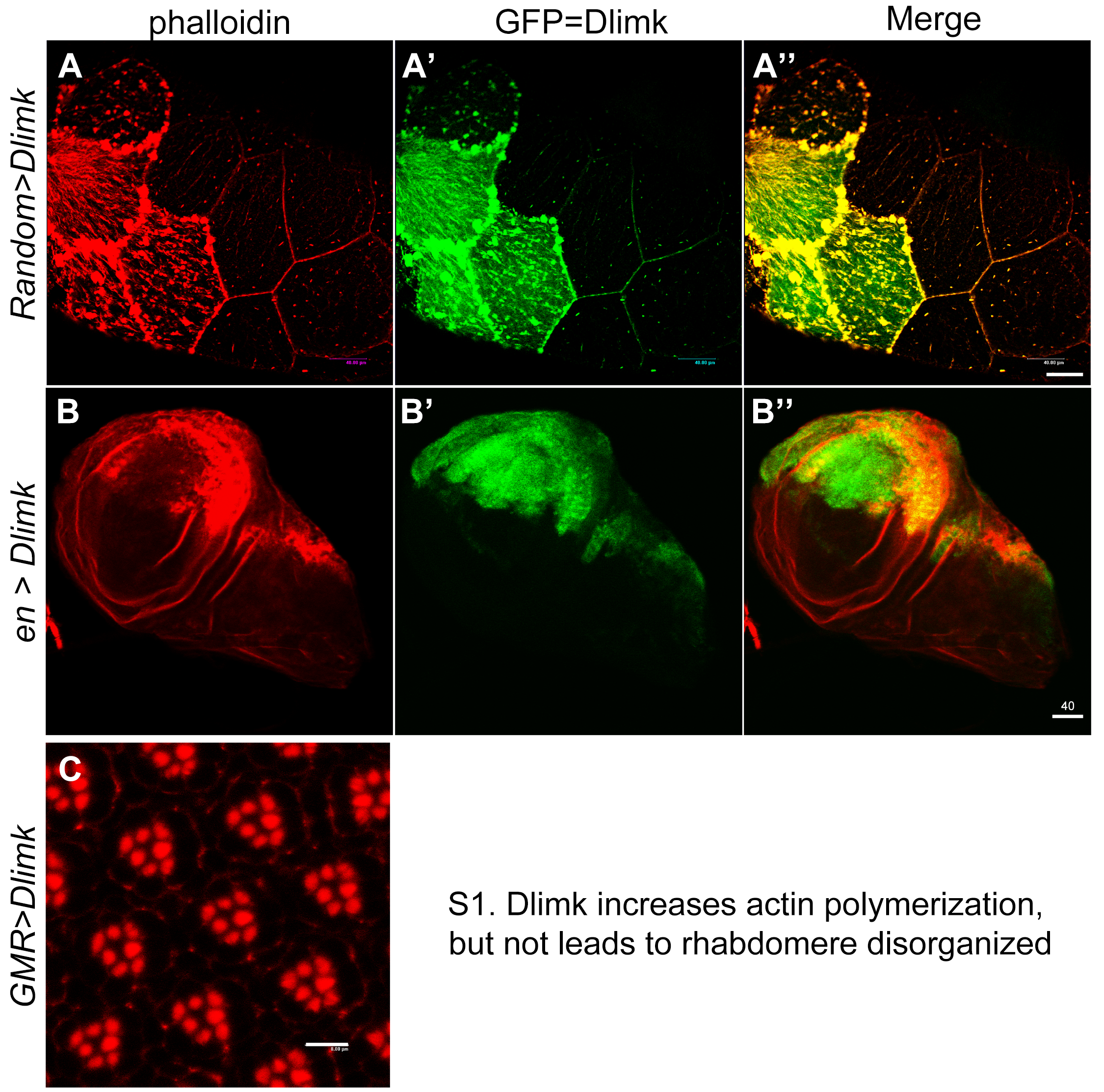

Supplement: S1 Fig — (A, B) Overexpression of Dlimk markedly raised the level of polymerized actin, revealed by random expression in the salivary gland or en-GAL4 in the wing disc. (C) Overexpressing Dlimk in eye did not disrupt the regular organization of rhabdomere. Green labeled cells represented GFP expression with Dlimk. Red florescence shows phalloidin staining. Scale bar, 40 μm (A”, B”), 8μm (C). Genetic background: (A) yw hsFLP; act>y+>GFP/ UAS-Dlimk. (B) en-GAL4, UAS-GFP/ UAS-Dlimk. (C) GMR-GAL4/ UAS-Dlimk. (TIF) [file pone.0186037.s001.tif]
